# A patient with the highly suspected B cell lymphoma accompanied by the erythrocytes cold agglutination: Case report

**DOI:** 10.1097/MD.0000000000034076

**Published:** 2023-06-23

**Authors:** Huijun Lin, Dujin Feng, Shuting Tao, Jianguo Wu, Yan Shen, Weizhong Wang

**Affiliations:** a Laboratory Medicine Center, Department of Clinical Laboratory, Zhejiang Provincial People’s Hospital, Affiliated People’s Hospital, Hangzhou Medical College, Hangzhou, Zhejiang, China.

**Keywords:** case report, 37°C-incubation, cold agglutinins, complete blood count, microscopic observation

## Abstract

Cold agglutinins are related with B cell lymphoproliferative disorder and lymphoma, and can agglutinate red blood cells (RBCs) at an optimum temperature of 3–4°C, which is the undergoing cause of RBCs cold agglutination. RBC cold agglutination may lead to an extreme abnormality of RBC parameters of complete blood count (CBC).

**Patient concerns:**

The present study reports a case of an old patient with severe infectious fever and anemia presenting extremely abnormal levels of RBC parameters in CBC and a sand-like appearance of blood on tube wall. The validating tests indicated the presence of the RBCs cold agglutination and the highly suspected B cell lymphoma.

**Diagnoses:**

The 37°C-incubation corrected the CBC results of the patient, and the microscopic observation and flow cytometry analysis of blood and marrow indicated many abnormal B lymphocytes. Subsequently, the patient was diagnosed with a highly suspected B-cell lymphoma.

**Interventions:**

The blood with a sand-like appearance was reanalyzed to validate the cold agglutination by 37°C-water incubation. The smears of peripheral blood and marrow were made for morphological observation by using optical microscopy. Moreover, the clusters of differentiation of the white blood cells were analyzed to confirm the type of abnormal white blood cells with a flow cytometer.

**Outcomes:**

The RBCs cold agglutination was validated, and the highly suspected B cell lymphoma was proved as the undergoing cause.

**Lessons:**

This case focuses on the discovery and solutions of RBCs cold agglutination, and emphasizes the importance of microscopic observation in the exploration of undergoing causes of cold agglutination.

## 1. Introduction

Cold agglutinins (CAs) are autoimmune antibodies, mainly immunoglobulin M (IgM), and are directed against the surface antigens of red blood cells (RBCs) at a thermal optimum of 4°C (thermal range 20–24 °C).^[1]^ CAs are able to agglutinate RBCs at an optimum temperature of 3 to 4°C,^[2]^ and are one of the common interference factors for RBC parameters of complete blood count (CBC). Due to RBCs agglutination, relevant RBC parameters of CBC generated by automated hematology analyzers can be significantly influenced. Although there are a few methods to eliminate CAs interference for CBC, 37°C-incubation is still one of the most widely used approaches to date.^[3]^ Production of CAs is from 2 types of conditions including chronic cold agglutinin disease (CAD) and secondary cold agglutinin syndrome (CAS).^[4]^ Primary CAD is caused by lymphoproliferative disorder of B lymphocytes,^[5]^ and it is distinguished from secondary CAS caused by some virus infections and malignancies.^[6]^ This article aimed to describe a case, where the patient had the highly suspected B cell lymphoma with severe RBC cold agglutination and anemia.

## 2. Case presentation

**Figure 1.**
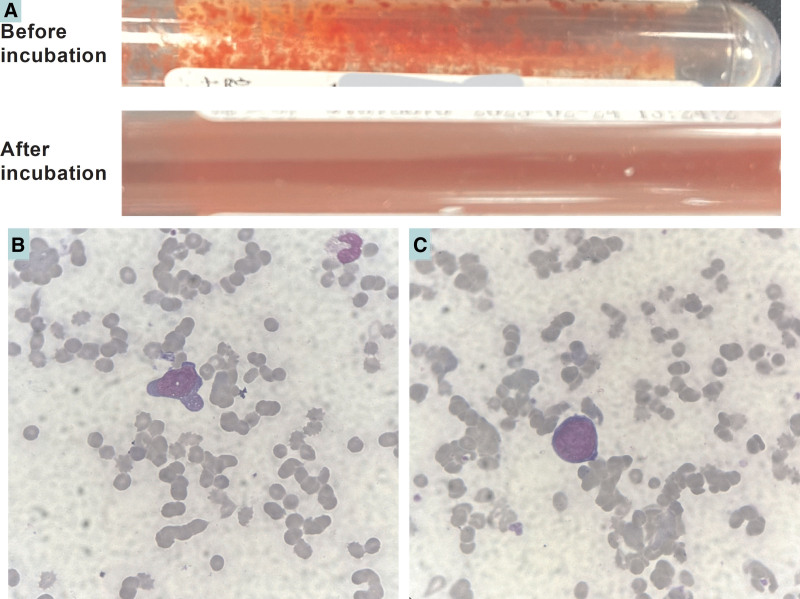
The blood appearances on the interior tube wall and cell morphology of blood smear. (A) shows the sand-like (above) and normal (below) appearances of the blood before and after 37°C-incubation, respectively. (B and C) show the normal RBCs and abnormal lymphocytes under different microscopic fields (x 1, 000), and the cytoplasm and nucleus of the lymphocytes were between normal and immature cells. RBCs = red blood cells.

A 67-year-old male patient was admitted with “chills and fever for 15 days,” and the admitted diagnosis was infectious fever, severe anemia, and thrombocytopenia. A computerized tomographic scanning examination revealed a “right adrenal block.” The patient was treated with meropenem immediately after admission, but he soon developed infectious shock and multiple organ failure and was then transferred to the intensive care unit for further treatment. When reviewing the patient’s report of complete cell count (CBC), we found that the parameters were characterized by a remarkable disproportion between RBC count (0.29 × 10^12^/L) and hemoglobin (Hgb, 60 g/L) level, a significant decrease in hematocrit (Hct, 2.9%), and an extreme elevation in mean corpuscular hemoglobin (MCH, 206.4 pg) and MCH concentration (MCHC, 2048 g/L), which indicated the obvious inaccuracy results (Table 1). When checking the analysis information on blood cell analyzer (Sysmex XN B1, Kobe, Japan), it showed the flags of “anemia, thrombocytopenia, red cell agglutination, and some atypical lymphocyte,” and so on. By careful observation of the specimen, we found that the blood on the interior tube wall was sand-like (Fig. 1A above), and speculated it might be a RBCs cold agglutination. After the specimen was incubated in a 37°C-water bath for 15 minutes, the sand-like blood aggregates could not be seen again (Fig. 1A below). Then we rerun the analysis using the same analyzer, and the CBC exhibited that MCH and MCHC returned to nearly normal levels, and the analyzer did not display any information on RBCs agglutination. The changes in the CBC results suggest that the patient might have had a cold agglutination phenomenon. Moreover, the subject also exhibited severe anemia and thrombopenia with an extremely high concentration of plasma high-sensitive C reactive protein of 166.7 mg/L.

**Table 1:** Results of peripheral CBC before and after bath incubation at 37°C.

Parameters	Original results	After incubation
RBC, x 10^12^/L	0.29	1.52
Hgb, g/L	60	55
Hct, %	2.9	15.8
MCV, fl	100.8	103.9
MCH, pg	206.4	36.2
MCHC, g/L	2048	348
RBC/Hgb, g^−1^	0.0048	0.0276
RBC/Hct, (L.%)^−1^	0.100	0.096

Data were analyzed by a hematologic cells analyzer.

CBC = complete blood count, Hct = hematocrit, Hgb = hemoglobin, MCH = mean corpuscular hemoglobin, MCHC = MCH concentration, MCV = mean corpuscular volume, RBC = red blood cell.

**Figure 2.**
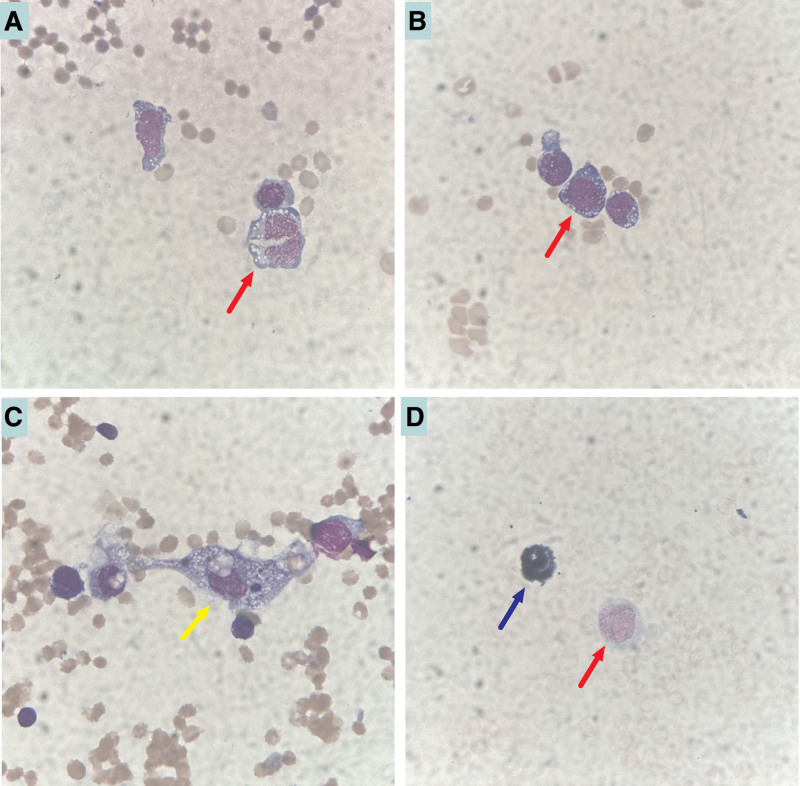
The cell morphology of bone marrow smears by oil microscopic observation (x 1, 000). (A–C) Wright-Giemsa staining. (D) Peroxidase staining. (A and B)Abnormal lymphocytes can be seen, and they accounted for about 18%. The cells are round or oval about 12–20 µm in size, partly irregular with a dragging tail and round nucleolus, as well as nuclear chromatin with 1–3 nucleoli, partly unclear, and the cytoplasma presents as blue with a moderate volume, and part of the cytosol shows visible vacuoles (marked with yellow arrow). (C) Hemophagages are also seen in the smear (marked with yellow arrow), accounting for about 1.5%. After (D) POX staining, a positive (marked with blue arrow) and negative (marked with red arrow) reaction were found in neutrophil and abnormal lymphocyte, respectively.

In order to find out the undergoing causes, blood smears were made using the specimen after 37°C-incubation. Then the smears were stained with Wright-Giemsa dye solution and were observed by oil microscopy. The morphology of RBCs showed no obvious abnormalities, no RBCs agglutination, and fragmented RBC, which did not present the characteristics of intravascular hemolysis. However, we found some abnormal lymphocytes on blood smear. Unlike the normal mature lymphocytes, the cytoplasm and nucleus of these cells seem to somewhere be between normal and immature cells (Fig. 1B and C). More importantly, the patient also exhibited an extremely increased lactate dehydrogenase activity (3512 U/L). Based on this finding, we suspected the possibility of lymphoma. Thus, we contacted the clinical doctors in chief and suggested a specific lymphoma might cause the cold agglutination, and bone marrow (BM) puncture was necessary for the primary diagnosis. Later results of BM observation suggested a lymphoma invasion in BM with hemophagocytosis (Fig. 2A–D). Next flow cytometry (FCM) analysis confirmed the subtype of lymphocytes and indicated a percent of about 16% and 18% of abnormal B lymphocytes in peripheral blood and BM, respectively, which strongly suggests a B cell lymphoma. Unfortunately, because the patient’s condition greatly worsened, and the patient’s relatives requested to be discharged, we did not perform further BM biopsy to confirm whether it was lymphoma.

Given that the patient was a subject with B cell lymphoma, it might lead the possible positive CAs, but we did not carry out the CAs test. Because CAs majorly are IgM-type autoantibodies, we further quantified the concentrations of serum Igs with an immunofixed electrophoresis assay. We observed nearly normal levels of IgG (10.3 g/L), IgA (1.15 g/L), and IgM (0.86 g/L), indicating a normal level of IgM-type antibody. However, a decreased serum albumin level (27.4 g/L) and a normal serum globulin (21.4 g/L) showed the increment of globulin/albumin ratio, revealing the relative increment of Igs. Moreover, significantly decreased levels of complement 3 (C3, 0.30 g/L) and C4 (0.04 g/L) were also found, which indicated a possible complement depletion due to the hemolysis.

Other laboratory assays were also performed. The cluster of differentiation (CD) analysis of peripheral blood by FCM showed negative results of CD7, CD13, CD33, CD38, CD117, and HLA expression, but some positive expressions (2+) of CD19, HLA-DR, CD20, CD200, CD43, CD22, and cyKi67 were found, which indicated the B cell subtype of lymphocytes. Moreover, biochemical analysis showed elevated levels of total bilirubin (TBil, 219.8 μmol/L), direct bilirubin (DBil, 201.7 μmol/L), indirect bilirubin (IBil, 18.1 μmol/L), suggesting possible extravascular hemolysis.

## 3. Discussion

The forms of CA (16–32% of the cases), the IgM class, are directed against the self-antigens of System I and activated at a thermal optimum of 4°C.^[1]^ Under pathological conditions, CAs directing against the RBCs may cause the consequent hemolysis, which is defined as autoimmune hemolytic anemia.^[7]^ RBC agglutination can cause a normal Hgb concentration with decreased RBC counts and Hct, and a marked elevation of MCH and MCHC levels, thus leading to clinically unreliable results. As early as 1945, Finland et al^[8]^ proposed incubation at 37°C as a method to eliminate CAs interference in CBC, thus, 37°C-incubation was used as the common approach to eliminate CAs interference compared to that of plasmapheresis, predilution, and double warming of specimens and reagents.^[3]^ According to the recognized rules of CAs interference, in the present case, the RBC parameters and flags of the CBC analyzer have indicated the possible RBCs agglutination in the specimen, and the visual evidence on the interior wall of the original specimen tube further revealed the high possibility of cold agglutination. After we carried out the incubation at 37°C, we were able to conclude the presence of RBCs cold agglutination in this blood specimen. We immediately rerun the analysis by using the incubated blood specimen at 37°C and obtained accurate results of all CBC parameters because the analyzer displayed no information on RBCs agglutination, and no RBCs agglutination was found by the subsequent microscopic observation. Therefore, this case suggests that, as experienced, skilled, and responsible laboratory consultants, we should find the clues of RBCs cold agglutination in time and correctly identify it from other agglutinations, then take effective approaches to obtain accurate and reliable RBC parameters.

Growing evidence supports the classification of CAD as a well-defined clinicopathological entity characterized by a clonal, low-grade lymphoproliferative BM disorder that results in the production of monoclonal autoantibodies, known as CAs.^[9]^ In contrast to CAD, secondary CAS, a similar cold hemolytic syndrome that usually complicates specific infections (Mycoplasma pneumoniae pneumonia or Epstein–Barr virus infection) or malignancies (especially aggressive non-Hodgkin lymphoma).^[10,11]^ Clinically, CAs are present in a high proportion of the adult subjects without the evidence of hemolysis or other underlying diseases, and these CAs are polyclonal and in low titers (<1:64), and monoclonal CAs seem to have higher titers and associated with hemolysis,^[11]^ which is uncommon, but also is one of the autoimmune hemolytic anemias characterized by the production of CAs directed against surface antigens on RBCs.^[6]^ Therefore, some patients with high titers of CAs and cold agglutination will probably present as the manifestation of hemolysis and anemia, and lymphoma may be one of the underlying causes. This case indicated a marked cold agglutination accompanied by severe anemia with a 60 g/L Hgb concentration and a significant increment of serum total and direct bilirubin, which strongly proved the evidence of extravascular hemolysis, and partly revealed the cause of severe anemia. Although CAD or CAS sometimes may cause intravascular hemolysis, it is uncommon. We also did not find any fragmented RBC on blood smear, which was not consistent with the typical laboratory performance of intravascular hemolysis, but presented the characteristics of extravascular hemolysis, and under this condition, the RBCs binding to antibodies are destroyed by the monocytes/macrophages system in liver or spleen. More importantly, the patient exhibited an extremely increased LDH activity (highly suggesting malignancies of lymphatic system), and we also found many abnormal lymphocytes on the blood and BM smear under microscope, and these cells were identified as abnormal B lymphocytes by flow cytometry with high percentage. After observation of BM smear, lymphoma-invasion in BM with hemophagocytosis was suggested by the blood morphology specialist, which further indicated a possible B cell lymphoma, the underlying cause of RBC cold agglutination, and the severe anemia of this patient. However, due to the worsening condition of the patient, we did not obtain a final diagnosis.

Although appropriately quality-controlled, good performance of hematology analyzers and properly operated them are sure to generate accurate CBC results for the vast majority of the specimens, almost every laboratory can encounter a few inaccurate results for some specimens. In CBC, specimen check and microscopic observation play important roles in accurate results of some abnormal specimens. In practice, we should carefully review each result and find underlying causes leading to inaccurate results. When RBCs count and Hgb concentration ratio in the CBC are significantly abnormal, extremely reduced Hct and increased MCH and MCHC are generated by analyzer, and the blood in the interior tube wall presents as a sand-like appearance under original condition, it strongly suggests the cold agglutination. The appropriate and simple approach is to incubate the specimen in a 37°C-water bath, then rerun the analysis. However, some specimens with high titer of CAs can not be corrected after 37°C-incubation, a higher temperature such as 41°C would be effective.^[12]^ For cold agglutination, besides correcting the results, we should also pay more attention to morphological examination, and timely observe the morphological characteristics of blood or BM cells, which will be one of the important approaches to find out some underlying causes of cold agglutination. Moreover, it is very necessary to actively discuss and communicate with the clinicians, so as to provide good suggestions for accurate clinical diagnosis and treatment.

## 4. Conclusion

In practice, it is important for the timely discovery and effective handling of RBCs cold agglutination, and microscopic observation is proven a practical and useful approach in the exploration of undergoing causes of cold agglutination and relevant anemia, which can provide a rapid and reliable laboratory suggestion for clinical diagnosis and treatment.

## Acknowledgments

We sincerely thank Prof Xianming Fei (Department of Clinical Laboratory, Zhejiang Provincial People’s Hospital) for his extremely important comments and suggestions during the design and writing of the manuscript.

## Author contributions

**Conceptualization:** Huijun Lin, Dujin Feng, Weizhong Wang.

**Data curation:** Huijun Lin, Dujin Feng, Jianguo Wu, Yan Shen.

**Funding acquisition:** Weizhong Wang.

**Investigation:** Huijun Lin, Dujin Feng, Shuting Tao, Jianguo Wu, Yan Shen.

**Project administration:** Huijun Lin, Dujin Feng, Jianguo Wu.

**Methodology:** Dujin Feng, Shuting Tao, Yan Shen.

**Resources:** Huijun Lin, Dujin Feng, Shuting Tao.

**Supervision:** Weizhong Wang.

**Validation:** Huijun Lin, Weizhong Wang.

**Writing – original draft:** Huijun Lin.

**Writing – review & editing:** Huijun Lin, Weizhong Wang.
